# Recent *N-*Atom Containing Compounds from Indo-Pacific Invertebrates

**DOI:** 10.3390/md8112810

**Published:** 2010-11-10

**Authors:** Yoel Kashman, Ashgan Bishara, Maurice Aknin

**Affiliations:** 1 School of Chemistry, Tel Aviv University, Tel Aviv University, Ramat Aviv 69978, Israel; 2 Laboratorie de Chimîe des Substances Naturelles et des Aliments, Faculté des Sciences et Techniques, Université de Réunion, 15 Avenue Rene Cassin, B.P. 7151, 97715 Saint Denis, Cedex 9, France

**Keywords:** heterocycles, *Fascaplysinopsis* sp., marine invertebrates, nitrogenous macrolide, leukemia cells

## Abstract

A large variety of unique *N-*atom containing compounds (alkaloids) without terrestrial counterparts, have been isolated from marine invertebrates, mainly sponges and ascidians. Many of these compounds display interesting biological activities. In this report we present studies on nitrogenous compounds, isolated by our group during the last few years, from Indo-Pacific sponges, one ascidian and one gorgonian. The major part of the review deals with metabolites from the Madagascar sponge *Fascaplysinopsis* sp., namely, four groups of secondary metabolites, the salarins, tulearins, taumycins and tausalarins.

## 1. Introduction

This report is an update of an earlier report of recent *N-*atom containing compounds [1] isolated by us between 2007 and 2010. The report includes organisms collected near Tanzania and along the west coast of Madagascar; the major part of the report deals with four groups of compounds isolated from the Madagascar *Fascaplysinopsis* sp. sponge. Our investigation of marine invertebrates started in 1972 on Red Sea soft corals, sponges and tunicates. The first *N-*atom containing compound isolated by us was a *N*-acyl-2-methylene-β-alanine derivative, isolated from the sponge *Fasciospongia cavernosa* [2].

To start with, there is the issue of the definition of alkaloids; namely, are all *N-*atom containing compounds alkaloids? The historical definition of “alkaloids” was: basic (alkaline like) plant derived, amino acid, metabolites. During the years, the term was also used for animal metabolites, well known for example, are frog alkaloids; furthermore, often the *N-*atom is a non-basic amide moiety. On the other hand, other basic compounds were defined as biological compounds or polyamines. The most common current definition was given in S.W. Pelletier‘s Alkaloids book [3] namely, “a cyclic organic compound containing nitrogen in a negative oxidation state which is of limited distribution among living organism”. There are quite a few unclear issues, such as, has the nitrogen atom to be part of the ring (is it required to be a heterocyclic one)? do cyclic peptides and depsipeptides belong? *etc.* On the other hand, the term *N-*atom containing compounds seems to be too broad, not focused enough [4].

**Figure 1:**
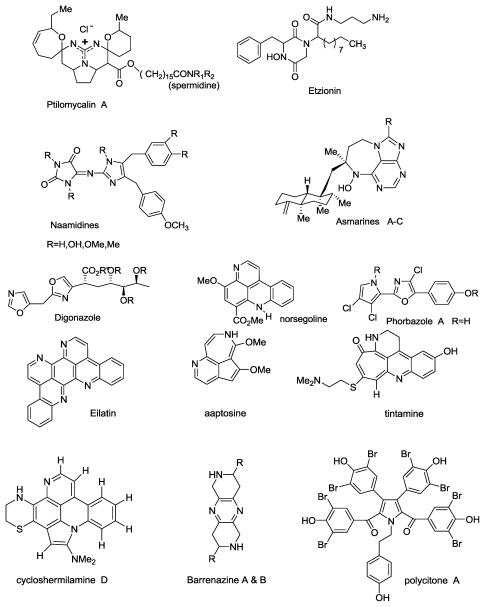
An assembly of unique marine alkaloids isolated by us.

An assembly of more special *N-*atom containing compounds, isolated by us over the years, is depicted in Figure 1.

Cyclic peptides are an example of compounds for which the term alkaloid is not usually applied. One example would be the *Didemnum molle* cyclic peptides.

**Figure 2:**
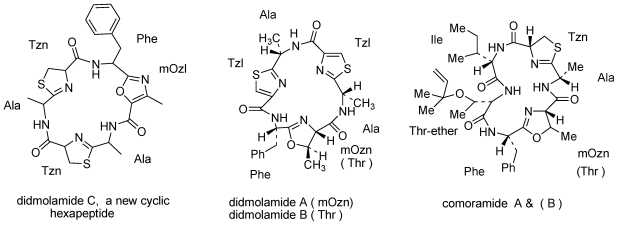
Cyclic hexapeptides isolated from *Didemnum molle*.

Ascidians of the genera *Lissoclinum* and *Didemnum* are prolific producers of cyclic peptides [5], many of which incorporate modified amino acid residues containing thiazole, oxazole, thiazoline or oxazoline rings. Similar structures have been reported from cyanobacteria [6], which led to the suggestion that the ascidian peptides were produced by symbiotic cyanobacteria. Among the many interesting cyclic peptides isolated from *D. molle* are comoramides A and B and didmolamide A and B (Figure 2) [7]. From yet another *D molle*, collected in Madagascar, we isolated didmolamide A and B and a new congener designated didmolamide C (Figure 2) differing from A and B in the oxidation state of the heterocyclic rings [8].

A major justification for exploring marine natural compounds are their unique structures with no terrestrial counterparts, many of which possess interesting biological activities, thus becoming excellent drug leads. Three examples from our earlier isolated compounds, namely, eilatin, violatinctamine and callynormine A [9–12] follow.

**Scheme 1:**
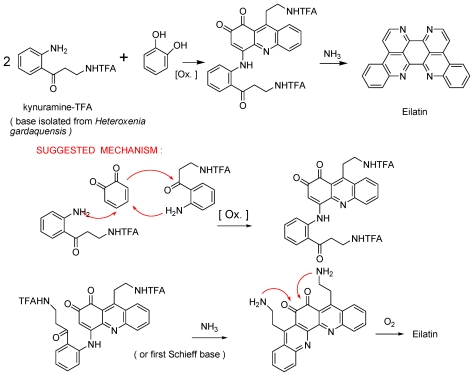
Biomimetic two steps synthesis of eilatin.

Among the more special heterocyclic compounds that we have isolated, is the symmetric tetraaza-heptacyclic compound, eilatin (Figure 1) [9]. Most intriguing was the biogenesis of eilatin that can be looked at as a bis-pyridoacridine. A suggested biosynthesis is depicted in Scheme 1. Indeed, eilatin could be prepared in two steps from two marine available metabolites, kynuramine and chatechol [9,10].

**Scheme 2:**
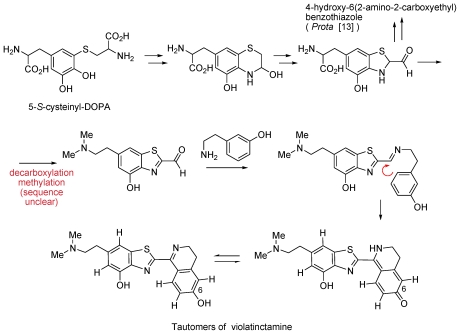
Tautomers of violatinctamine.

The biogenesis of violatinctamine, another interesting alkaloid, is shown in Scheme 2. Violatinctamine, a new alkaloid, together with four other known metabolites were isolated from the tunicate *Cystodytes cf. violatinctus* collected in Kenya [11]. Violatinctamine has a unique heterocyclic skeleton which combines a benzothiazole unit and a dihydroisoquinoline unit (Scheme 2) [13]. The structure of violatinctamine was elucidated by interpretation of MS as well as 1D and 2D NMR spectra (Scheme 2) [11].

Benzothiazoles rarely occur as marine natural products. The first benzothiazoles from the marine biosphere were isolated from fermentation culture extracts of *Micrococcus* sp., a marine bacterium obtained from the tissues of the sponge *Tedania ignis* [14]. The latter compounds included 2-mercaptobenzothiazole, 2-methylbenzothiazole, 2-hydroxybenzothiazole and 6-hydroxy-3-methyl-2-benzothiazolone. Another benzothiazole derivative designated S1319 was isolated from *Dysidea* sp. and exhibited bronchodialating activity [15].

**Figure 3:**
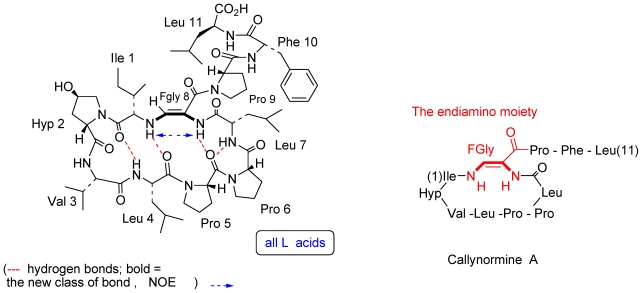
Callynormine A, a new type of cyclic peptide.

The third example is that of callynormine A (Figure 3), the first compound of a new class of heterodetic peptides embodying the α-amino-β*-*aminoacrylamide functionality (instead of the ester group of depsipeptides) which we named cyclic endiamino peptides [12].

**Scheme 3:**
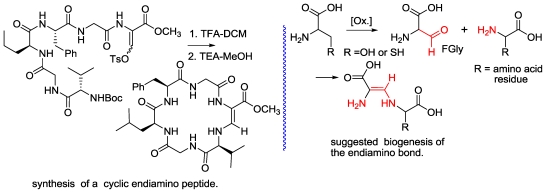
Suggested biogenesis of the endiamino bond and the synthesis of an endiamino cyclichexapeptide.

**Scheme 4:**
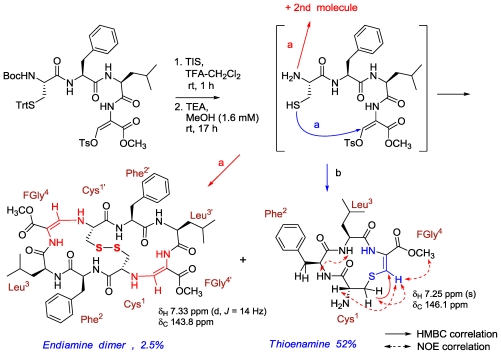
The synthesis of cyclic endiamino and thioenamino peptides.

It is suggested that the endiamino group is derived from the condensation of the formyl group of FGly and the amino group of another amino acid (Ile in the case of callynormine A) (Scheme 3) [12]. Formylglycine (FGly) was reported for both eukaryotic and prokaryotic sulfatases located within the catalytic site of the enzyme [16,17]. It was shown that the formylglycine is generated by oxidation of cysteine or serine and, furthermore, that the FGly hydrate is covalently sulfated, or covalently phosphorylated during catalysis. To the best of our knowledge, there are no reports of natural compounds embodying the endiamino group. Indeed, synthetic linear compounds with this group are known. The endiamino group is of special interest for the synthesis of biomimetic cyclic peptides, as it is expected to introduce additional rigidity into their structure. We demonstrated the synthesis of several cyclic endiamino peptides, including 2-(1*H*)-pyrazinone, which, formally, is the smallest member of this new group [18]. We also demonstrated the preparation of endiamino containing building blocks for biomimetic peptides [18]. FGly is very unstable. However, its enol-tosylate derivative, prepared from serine, is stable and acts with amino groups as an aldehyde [19] to produce the α-amido-*β*-aminoacrylamide functionality. As mentioned above, novel marine natural products serve as drug leads and more specifically, in the endiamino case, are a lead for cyclic endiamino peptides (Scheme 3 and 4). Furthermore, it could be shown that instead of the nucleophilic amino group attack on the tosyl enolate, the superior thiol can be used, thus, generating another new group of compounds, the thioenamino cyclic peptides [20] (Schemes 3 and 4).

## 2. Results and Discussion

New *N-*atom containing compounds isolated by us during the last three years.

### 2.1. Salaramides A and B

**Figure 4:**
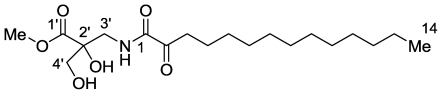
The structure of salaramide A and B (the 15-homologe).

From the Madagascar sponge *Hippospongia* sp. phylum Porifera, class Demospongiae, order Dictyoceratida, family Spongiida, we have isolated two new α-oxoamides, designated salaramide A (main compound) and its higher homologue salaramide B (minor compound) as an inseparable mixture (Figure 4) [21].

α-Oxoamides have not been reported, to the best of our knowledge, in marine sponges and never found in the genus *Hippospongia*. The structures of the compounds were established from the HRESIMS and 1D and 2D NMR experiments [21]. Interestingly, acetylation of salaramide A, under the usual mild conditions, resulted in a triacetate due to acetylation, in addition to the two hydroxyls, of the enol-hydroxyl of the α-oxoamide. The transformation of an α-ketoamide to enolacetate is rare, but was reported earlier for an α-oxolactame [21]. From a biogenetic point of view, the salaramides are unique aminodihydroxyisobutyric acid derivatives, *i.e.*, the amino analogues of the naturally rare trihydroxyisobutyric acid.

### 2.2. Plakinamine L

**Figure 5:**
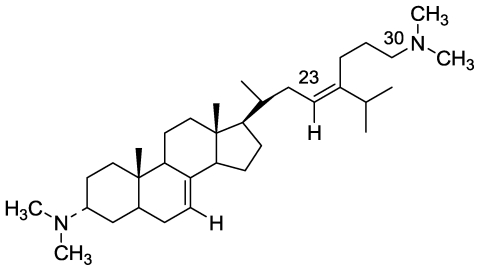
The structure of plakinamine A.

The marine sponge *Corticium* sp. represents a prolific source of steroid alkaloids [22,23], the majority of which are structurally related to plakinamine A [24], as they possess an amino group at C-3 and a cyclic amine functionality on the side chain of a C29 steroidal framework. From the Madagascar *Corticium* sp. sponge we have isolated a congener of plakinamine A designated plakinamine L (Figure 5) [25]. Plakinamine L was assigned the molecular formula C_33_H_58_N_2_ by combined HREIMS and ^13^C NMR analyses, and its structure elucidated by 1D and 2D NMR spectra as well as comparisons with known plakinamines [25]. Plakinamine L, is the first member of the group with an acyclic side chain.

### 2.3. Saldedines A and B

**Figure 6:**
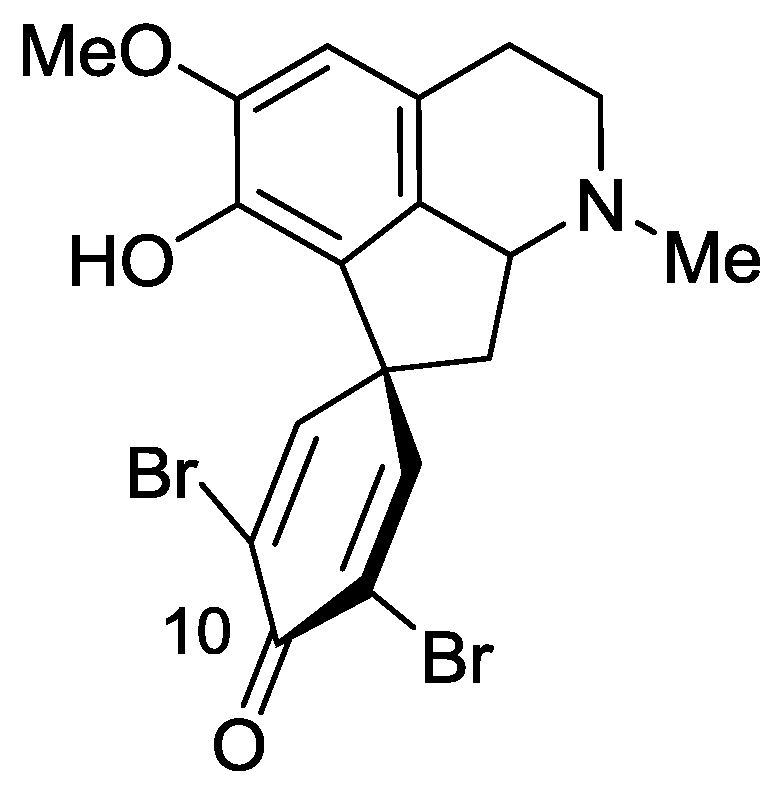
The structure of saldedines A and B (10-OH).

Two new dibromo proaporphine alkaloids, designated as saldedines A and B, were isolated from an unidentified Didemnidae tunicate, collected in Salary Bay, north of Tulear, Madagascar [26]. Plant proaporphine alkaloids, a major isoquinoline group, have been recognized as the biosynthetic precursors of aporphine alkaloids bearing a wide range of oxygenated substitution patterns with mainly a spiro-cyclohexadienone ring system (Figure 6). These alkaloids have, so far, only been isolated from a species of the Papaveraceae plant [27].

Several of these alkaloids exhibit interesting biological activities [28]. To the best of our knowledge the present work is the first report of proaporphine alkaloids from a marine source, and for the first time of bromo proaporphines. As in many other cases, the real source of these compounds, the tunicate or guest microorganism, is unknown. The structure elucidation of the two new compounds was achieved by HRESIMS and 1D and 2D NMR spectra [26]. Finally, a qualified single crystal was obtained from CH_2_Cl_2_, enabling X-ray crystallographic analysis (Figure 6) and confirming unequivocally the structure of saldedine A. Interestingly, saldedine A crystallized as a racemic mixture in space group *Pbca*, wherein the two enantiomeric species are related by an inversion center.

The biosynthesis of proaporphine alkaloids is derived in nature (plants) from oxidative phenolic coupling of the benzylisoquinoline alkaloide. Whereas proaprophines are not known from marine sources a possible benzylisoquinoline precursor was previously obtained from the starfish *Dermasterias imbricata* [29].

Saldedines A and B were both tested for toxicity to brine shrimp (*Artemia salina*) and were found moderately active. Saldedine A shows a greater potency with a LD_50_ value of 4.4 *μ*M, while saldedine B has a LD_50_ value of 10.9 *μ*M.

### 2.4. Njaoamines G and H

A variety of sponge extracts were screened for brine shrimp toxicity. As a consequence, we encountered potent activity from a *Neopetrosia* sp. collected off Pemba Island, Tanzania. Bioassay-guided isolation of the active compounds resulted in the isolation of two new polycyclic alkaloids designated njaoamine G and H [30], belonging to the njaoamine‘ family, represented by njaoamines A–F, isolated recently from a haplosclerid sponge *Reniera* sp. [31]. The structure elucidation of njaoamine G (and H) was achieved by MS and 1D and 2D NMR experiments. The MS and signals in the ^13^C NMR spectrum were consistent with the molecular formula C_39_H_52_N_4_ that was established by HRFABMS (m/z 577.8956, calcd for C_39_H_53_N_4_, 577.8959), suggesting 16 degrees of unsaturation. The thorough analysis of the COSY, TOCSY, HSQC and HMBC experimental data contributed to the structure of njaoamine G, a congener of the earlier reported njaoamines [31] and −H, both possessing a decan-4-yne chain. The stereochemistry of the alicyclic pentacyclic portion of the molecule was deduced from NOESY correlations and confirmed to be comparable to the njaoamines‘ and ingenamines‘ families [32–34]. Njaoamine H was shown to be the 9-hydroxy, quinoline, analog.

Njaoamine G and H were both tested for toxicity to brine shrimp (Artemia salina) and were found to be highly active. Njaoamine H showed greater potency with an LD_50_ value of 0.08 μg/mL; njaoamine G had an LD_50_ value of 0.17 μg/mL [30]. It is worth noting that njaoamines A–F showed cytotoxic activity against three human tumor cell lines [30]. Although the present metabolites are structurally close to the early reported alkaloids from the sponge Reniera sp. (njaoamines A–F) [31], they give an additional insight into the unique metabolic processes in their construction. The highly potent and exceptional brine shrimp toxic activity of njaoamine G and H will encourage us to continue the current investigation of their biological activity.

**Figure 7:**
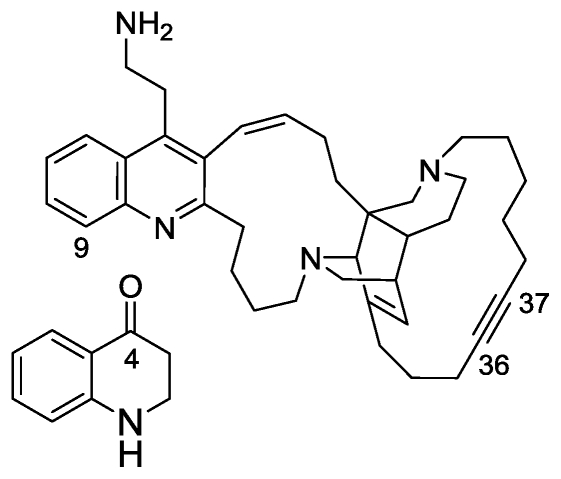
The structure of njaoamine G and H (9-OH) and 4-quinolone.

In addition to the two njaoamines, we also isolated from the sponge extract 1,2,3,4-tetrahydroquinoline-4-one (Figure 7), a plausible precursor of the njaoamines. Though this quinolinone is commercially available, to the best of our knowledge, this is the first report of its origin from a natural source.

### 2.5. Nuttingins A–F and Malonganenones D–H

The extract of the gorgonian *Euplexaura nuttingi* (Kukenthal, 1919) collected in Uvinage, Pemba Island, Tanzania, was found to possess a moderately active apoptosis-inducing activity. Positive alkaloid coloring of the extract of this horny coral suggested *N*-atom-containing metabolites. Gorgonians, well known for production of isoprenoids and polyketide metabolites [35,36], are poor in *N*-atom-containing compounds, and only a few such compounds are known [36]. A recent report on tetraprenylated purines, the malonganenones, which possess antiesophageal cancer activity, from the Mozambique gorgonian *Leptogorgia gilchrist* [37] encouraged us to report our findings from the Tanzanian gorgonian *E. nuttingi*.

**Figure 8:**
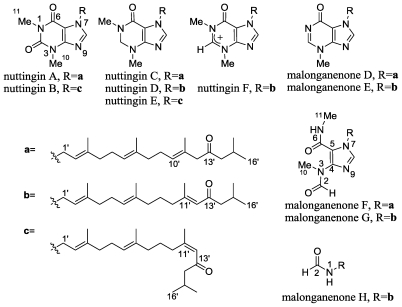
The structures of nuttingins A–F and malonganenones D–H.

Six new tetraprenylated purine alkaloids, designated nuttingins A–F, as well as eight malonganenones, tetraprenylated alkaloids, of which five (D–H) are new, and three (A–C) known, were isolated from this horny coral (Figure 8).

^15^N NMR can be a powerful tool for structure determinations of *N*-atom containing natural products [36–40]. The suitability of ^15^N NMR spectroscopy is attributed to the wide range of chemical shifts and its great sensitivity to structural and environmental changes. A major disadvantage of this spectroscopy is the extremely low sensitivity of ^15^N at the natural abundance level. However, the sensitivity issue can be overcome since inverse detection makes it possible to acquire one bond and long-range ^1^H-^15^N correlations, circumventing the low sensitivity, and therefore obtaining ^15^N chemical shifts. In previous papers we demonstrated the benefit of using ^15^N NMR data (NH correlations ^2^*J*_NH_ and ^3^*J*_NH_ HMBC) for the structure determination of various *N*-atom-containing compounds including purine derivatives [40,41].

The ^1^H-^15^N HMBC of nuttingin A unequivocally confirmed the structure of the proposed heterocyclic ring system [42]. ^2^*J*_NH_ correlations observed between H-8 and two vicinal nitrogen atoms resonating at 168.7 and 229.7 ppm established these nitrogens to be N-7 (pyrrole sp^3^) and N-9 (sp^2^). Additional ^2^*J*_NH_ correlations were observed between the two *N*-methyls to their neighbor *N*-atoms, *i.e.*, between Me-10 and a nitrogen atom resonating at 113.6 (N-3) and between Me-11 and a second nitrogen atom resonating at 150.5 ppm (N-1). Nuttingins C–E are unstable, as was concluded by monitoring their NMR spectra in CDCl_3_ or *d*_6_-DMSO. Within three to four days in the NMR tube, the purine system of the latter compounds changed into the cationic system found in nuttingin F. Characteristic of this oxidation were the down-field shifts of the two *N*-methyl signals.

Together with the nuttingins, three known malonganenones (A–C) [37] and five new ones (D–H) [42] were also isolated. The structures of the latter compounds (Figure 8) was determined by the comparison of their NMR data to the known compounds and MS and 2D NMR experiments [37,42].

Nuttingins A–E and malonganenones D–G have been found to inhibit growth of K562 [43] and UT7 [44] cells. The tests were done on mixtures of pairs of compounds, as there was no real difference between the activities of compounds differing only in the side chain. To investigate the potential effects of the compounds on cell proliferation, two different human leukemia cell lines, K562 and UT7, were used as targets and treated with various concentrations of the tested compound, for 24 and 48 h. shown in Nuttingins C-E induced inhibition of cell growth in K562 (A) and UT7 (B), in a dose- and time-dependent manner. UT7 cells displayed a greater sensitivity as compared to K562. Namely, at 0.4 *í*g/mL nuttingins C to E induced 50% inhibition of cell growth in UT7 cells (B) and 30% in K562 cells (A), after 48 h of exposure to the compounds. The rest of the new compounds also displayed inhibitory activity on proliferation of both cell lines, although they were approximately 3-fold less potent.

### 2.6. Isohalitulin and Haliclorensins B and C

**Figure 9:**
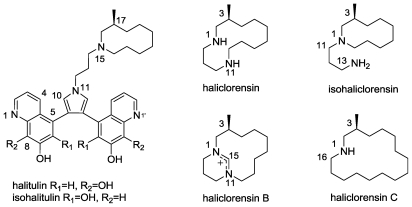
The structure of Haliclona tulearensis *N*-atom containing metabolites.

Halitulin and haliclorensin are two unique alkaloids isolated in our group from the marine sponge *Haliclona tulearensis* collected in Sodwana Bay, Durban, South Africa [45,46]. The significant cytotoxicity of haliclorensin against P-388 mouse leukemia cells and that of halitulin against several tumor cell lines has stimulated studies toward the total syntheses of both molecules [47–49]. Steglich and Banwell’s syntheses of haliclorensin [49] allowed the revision of its structure (Figure 9), and the initially assigned structure for haliclorensin was subsequently renamed isohaliclorensin [49,50]. Furthermore, it was suggested that both the initially proposed structure (the azacyclodecane precursor of halitulin) and the revised structure, originate from a common 1,11-diazabicyclo[8,4]tetradecane [47]. Two recent reports on the total synthesis of halitulin confirmed its structure and allowed the determination of its absolute (17*S*) configuration [47,50].

Together with halitulin and haliclorensin, an additional related compound was isolated from the same sponge [45,46]. Because this compound was isolated in minute amounts and was highly sensitive to light and air, the structure elucidation was not accomplished. As part of our ongoing search for novel bioactive substances from marine invertebrates, we resumed our work on *Haliclona* sponges. The constituents of two Madagascan *Haliclona tulearensis* sponge specimens were examined with the purpose of finding additional interesting metabolites and hopefully to, once again, isolate the above mentioned sensitive compound and complete its elucidation.

Indeed, the two new samples of *Haliclona tulearensis*, collected at Salary Bay, *circa* 100 km north of Tulear, Madagascar, contained three new alkaloids designated isohalitulin (the compound searched for) and haliclorensins B and C (Figure 9).

The structures of all three were elucidated by MS and 1D and 2D NMR spectra. Isohalitulin is a structural isomer of halitulin where a change of the hydroxyl position on the quinoline system occurs [51].

Haliclorensins B and C represent novel *N-*atom containing compounds [51]. To the best of our knowledge, the only naturally reported tetrahydropyrimidinium ring, as in haliclorensin B, is the pyrrole-derived alkaloid *N*-methylmanzacidin C isolated from the sponge *Axinella bre*V*istyla* [52]. Indeed, the tetrahydropyrimidinium ring by itself is known synthetically [53,54]. Haliclorensin C joins two other marine, naturally occurring azacycloalkanes, *i.e.*, keramaphidine C (6*Z*-azacycloundecene), the first reported marine azamacrocycle [55] and haliclorensin 7-methyl-1,5-diazacyclotetradecane. On the grounds of common biogenetic precursors, it is tentatively suggested that isohalitulin and haliclorensins B and C have the same absolute configuration of the single stereogenic center (*S*) as determined for halitulin and haliclorensin.

Obtaining different secondary metabolites from the two Salary Bay collections of *H. tulearensis* and from a sample collected on the other side of the Mozambique Canal raises the question of the real source of the compounds, namely, the sponge or guest microorganisms. Isohalitulin and haliclorensins B and C were tested for toxicity to brine shrimp (*Artemia salina*) and were found to be moderately active. Isohalitulin shows a greater potency, with a LD_50_ value of 0.9 mM, while haliclorensins B and C have LD_50_ values of 2.2 and 2.1 mM, respectively.

## 3. Introduction to the *Fascaplysinopsis* sp. Metabolites

In continuation of our investigation of Madagascar marine sponges, we have investigated the *Fascaplysinopsis* sp. sponge collected in Salary Bay *circa* 100 km north of Tulear. The identification of this spicule-less sponge genus by Professor Vacelet, Marseille, was not straightforward. It seems to be closest to *Fascaplysinopsis* (Demospongiae, order Dictyoceratid, family Thorectidae) a genus described thus far only from Australia and Indonesia.

**Figure 10:**
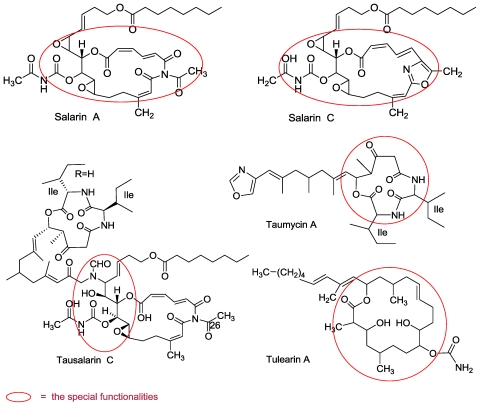
Salarins, tulearins, taumycins and tausalarins, four new groups of metabolites from the Madagascr sponge *Fascaplysinopsis* sp.

From this organism we have isolated and elucidated the structure of four groups of unprecedented cytotoxic nitrogenous macrolides *i.e.*, salarins (A–J) [56,57,61,62] tulearins (A–C) [56,59] taumycins (A and B) [58] and a fourth group, combining taumycin and salarin, designated tausalarins (C) (Figure 10) [60].

All four groups are novel classes of marine natural compounds embodying unprecedented structures with rare or even naturally unknown functional moieties. The structure similarity of the four groups to microorganism and fungal metabolites (e.g., to the cyanobacteria *Lyngbia bouillonii* metabolites, madangolide and laingolide A) [63,64], suggested that these compounds originate from guest microorganisms rather than from the host sponge itself. This notion is supported by the chemical content variations from one collection to the other. During the last four years we collected the sponge several dozen times from different spots in Salary Bay and found remarkable differences in their metabolites.

The extracts of the Madagascar *Fascaplysinopsis* sp. were found to be active in the brine shrimp test as well as cytotoxic to leukemia cells. All compounds were evaluated for their cytotoxicity against K562 [43] and UT-7 [44] human leukemia cells lines, using the colorimetric methylthiazole tetrazolium bromide (MTT) assay [62]. Salarin C was found to be the most potent compound, exhibiting significant inhibitory activity against the leukemia cell lines, UT-7 and K562, and the murine pro B cell line Ba/F3 at concentrations of 0.0005–0.5 μg/mL [62].

### 3.1. Salarins A–J

**Figure 11:**
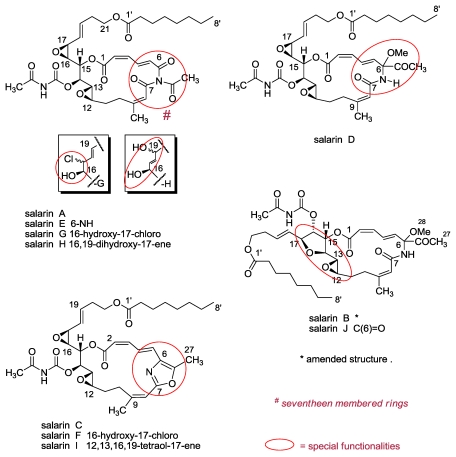
The ten *Fascaaplysinopsis* sp. salarins (A–J).

Salarin‘s A formula was determined from the mass spectrometric as well as the carbon-NMR analysis suggesting a molecular formula of C_35_H_46_N_2_O_12_ (HRESMS *m/z* 709.2991 for [M + Na]^+^, with 14 degrees of unsaturation. Comprehensive analysis of the 1D and 2D NMR data including the spectra of the *N-*methyl derivative, *vide infra*, and comparison with model compounds led to the suggested structure (Figure 11) [56].

Worth mentioning is the outstanding low field double doublet at δ 8.21 (*J* = 15.7 and 11.3 Hz), in moiety (d) (Figure 13) characteristic for a *2Z*, *4E* geometry. This exceptionally low field signal agrees only with the *2Z*, *4E* isomer and requires a carbonyl at position C-6 [65].

The naturally unique *N*-acetyl carbamate group of salarin A was suggested following CH- and NH-HMBC experiments (δ_N_ 143 ppm) [56] and was in good agreement with the acidity of the imide proton, among the two carbonyls, which could be methylated with CH_3_I, in the presence of K_2_CO_3_ in acetone, to afford the *N*-CH_3_ derivative (δ_H_ 3.23s, δ_C_ 30.3q).

**Figure 12:**
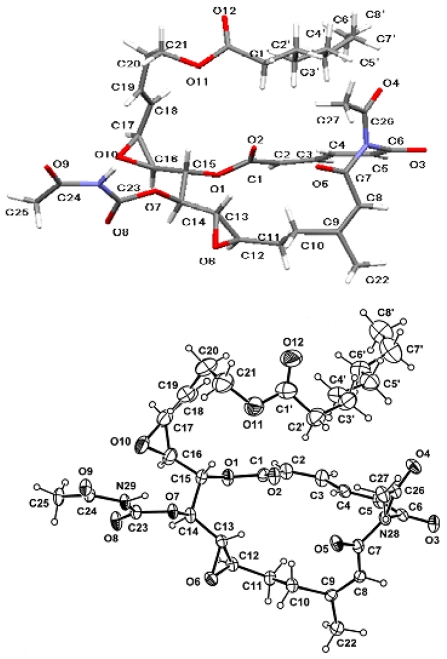
Wire frame model and ORTEP representation of salarin A, obtained by X-ray diffraction analysis.

After lengthy crystallization trials a crystalline structure of salarin A suitable for X-ray diffraction analysis was obtained and the relative configuration of the six chiral centers achieved [60] (Figure 12).

Salarin B analyzed for C_36_H_52_N_2_O_13_ from the HRESMS (*m/z* 741.3028 [M + K]^+^), with 13 degrees of unsaturation. The NMR data pointed to high similarity to salarin A, disagreeing only in three functional moieties. That is, -B lacks the 16,17-epoxide, differs in the C-14 to C-17 site, and also lacks the triacylamine group (f), *vide suppra*. Instead of the latter functionality, -B possesses, as part of the macrolide a lactam group carrying next to the nitrogen atom (on C-6), a methoxyl and a methyl ketone (Figure 11). The latter unique moiety resembles a similar rare functionality in the *Aspergillus* metabolite synerazol [66]. The structure of the C5–C9 segment was suggested on the basis of 2D NMR data.

**Scheme 5:**
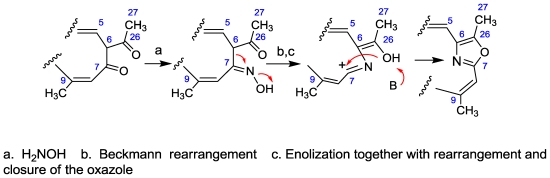
Suggested biogenetic of the oxazole ring of salarin C [63].

Both salarin A and B possess novel macrolide structures. Not only in the triacylamine and the α-substituted lactam functionalites, but also in the construction of the macrolide from two carbon chains (a 6-amidohexa-2,4-dienoic acid and a functionalized C_15_-carboxylic acid). It is also feasible that the nitrogenous macrolide is obtained by a Beckmann rearrangement of an α-ketooxime of a single chain, namely, introducing a nitrogen atom into the chain which can afford an amide or oxazole [67] (Scheme 5). A similar combination of two chains can be found in the two above mentioned nitrogenous macrolides madangolide and laingolide A [63] isolated from the cyanobacteria *Lyngbia bouillonii* [64].

**Figure 13:**
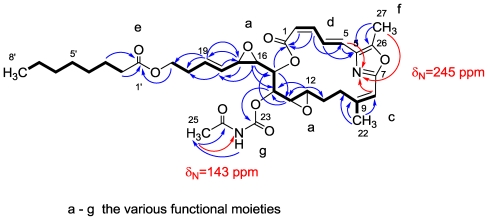
COSY (**—**), and key ^13^ CH- and ^15^NH-HMBC correlations of salarin C.

From yet another collection of the *Fascaplysinopsis* sp. sponge, we isolated several additional compounds including one bright orange in color which was closely related to salarins A and B and designated salarin C (Figure 13).

The mass spectroscopic analysis of salarin C provided a pseudo molecular formula of C_35_H_46_N_2_O_10_Na, HRESIMS, *m/z* 677.3035 for [M + Na]^+^ (calcd. 677.3044), with 14 degrees of unsaturation. The ^1^H, ^13^C, COSY, HSQC, TOCSY, and HMBC spectra [57] (Figure 13), established the structure of salarin C. The major change was the replacement of the triacylamine subunit of salarin A by an oxazole ring in salarin C, evidenced by the ^15^N resonance-value, measured from the ^3^*J*(C**H**-**N**) HMBC correlation, of δ 245.0 ppm [68] (in addition to the δ 143.0 ppm shift of the acetyl carbamate nitrogen atom) (Figure 13).

**Scheme 6:**
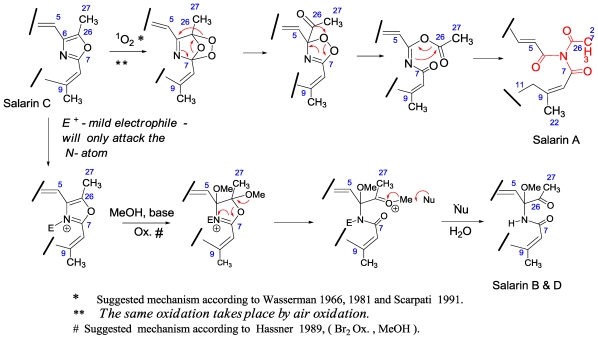
Suggested biogenetic transformations of salarin C to salarins A, B and D.

Oxazoles are widely present in biologically active natural compounds. It was believed that the oxazole rings were biosynthesized from amino acids, namely from serine or threonine. The amino acid origin of oxazoles in cyclic peptides such as bistratamide is most likely, and it has even been proven in epothilone D, for example [69]. However, oxazole precursors in the marine natural compounds calyculins, phorbazoles and mycalolide were recently proposed by Uemura to be obtained via a different route involving Beckman rearrangement of α-formyl ketoximes [67]. Similarly, it can be suggested that the oxazole ring of salarin C is obtained from an α-acetyl ketoxime as depicted in Scheme 5. Of prime interest was finding in the literature that oxazoles ring open under oxidative conditions to afford with ^1^O_2_ the triacylamine moiety [70] and under mild basic bromine-oxidation, an amidomethoxy ketone [71]. Salarins A and B are precisely the expected products from salarin C under analogous biosynthetic oxidations. Detailed suggested mechanisms leading to salarins A and B from C are depicted in Scheme 6.

Unexpectedly, stirring salarin C in chloroform slowly afforded salarin A (ca. 50% in 2–3 days; over a longer period, isomerization of the α,β,γ,δ-dienoate takes place). However, it was found that an efficient transformation of salarin C to -A, almost quantitatively, takes place when a thin layer of salarin C over glass, was left over night in the air under light.

The anti-proliferative activity of salarin C was dose-dependent; salarin C at concentrations of 0.0005–0.5 mg/mL was added to the cells for 24 hours, and cell viability was determined by MTT assay. Salarin C was by far more potent than salarins A and B and tulearin A [62]. In addition to the above salarins, we have isolated seven others (D–J) in minute amounts. The following structure elucidations/discussions are grouped according to their similarity to salarins A–C, the first group being salarins E, G and H (Figure 11).

Comparison of the ^1^H and ^13^C NMR data of salarin E [61] with those of salarin A [56], indicated high similarity; the major distinction being the absence of the 6-*N*-acetyl resonance, replaced by a NH singlet at 7.96 ppm. The chirality of the six stereogenic carbon-atoms is assumed to stay unchanged.

The HRESIMS spectrum of salarin G exhibited a pseudomolecular ion [M + Na]^+^ at *m/z* 745.2727 suggesting, together with the ^13^C spectrum, the molecular formula C_35_H_47_ClN_2_O_12_, implying 13 degrees of unsaturation. The presence of one chlorine atom in the molecule was further confirmed by two dominant sodiated pseudomolecular ions [M + Na]^+^ at *m*/*z* 745.3 and 747.3 with intensities of 1/0.33 in the ESIMS spectrum. Inspection of the ^1^H and ^13^C NMR features of salarin G closely resembled those of **-**A except for changes in the side chain. Namely, the 16(17)-epoxide is replaced by a 16-oxymethine-17-chloromethine functionality. Assuming salarin A to be the precursor of salarin G, it can be suggested that the original epoxide-oxygen atom retains its configuration while the configuration of the chlorinated allylic C-17 atom is unknown. To exclude the possibility that salarins G and F, *vide infra*, are artifacts, products of HCl opening of the epoxide of salarin A or C, we treated salarin A with traces of DCl in CDCl_3_ and monitored the proton NMR. It was found that salarin A is relatively stable under these mild acidic conditions for 48 h and then, as with higher concentrations of acid, affords complex mixtures and not a single chlorohydrin.

**Figure 14:**
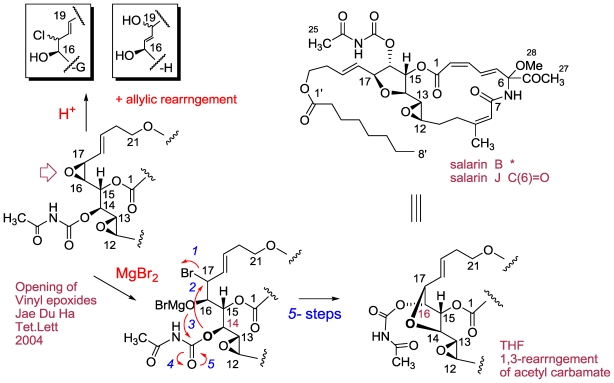
Transformations of salarin A and/or C.

Salarin H, the third congener of salarin A, possesses the formula C_35_H_48_N_2_O_13_, *i.e.*, addition of a molecule of water to salarin A. Comprehensive analysis of the NMR data, 2D experiments [61] and comparison with former congeners, established for salarin H the 16,19-dihydroxy-17-ene structure. Obtaining the latter moiety can be explained by acid catalyzed opening of the 16(17)-epoxide of **-**A, followed by allylic rearrangement of the initially obtained 18-ene-17-carbocation intermediate (Figure 14).

A congener of salarin B is salarin D exhibiting a pseudomolecular ion [M + Na]^+^ at *m/z* 725.3205 suggesting, together with the ^13^C NMR data, the molecular formula C_36_H_50_N_2_O_12_, thus implying 13 degrees of unsaturation [61]. The 1D and 2D NMR spectra of -D revealed a close relationship with salarin B, that is, **-**D possessing the same methoxymethylketone lactam moiety (the C6–C8 segment); the major change being a 16(17)-epoxide in -D, in addition to the 12(13)-epoxide [56,57].

Two additional isolated congeners of salarin C are salarin F and I (Figure 11). Salarin F possesses the oxazole ring of -C and the 16-hydroxy-17-chloro-moiety of salarin G. Salarin I, a relatively polar compound, on the other hand, misses the two epoxides and possesses, in addition to the oxazole ring, a 12,13,16,19-tetraol-17-ene functionality [61].

The tenth salarin, salarin J, exhibited a pseudomolecular ion [M + Na]^+^ at *m/z* 667.2827, suggesting, together with the ^13^C NMR spectrum, the C_33_H_44_N_2_O_11_ molecular formula, indicating 13 degrees of unsaturation. Hence, salarin J was implied to be a structural isomer of **-**D, differing according to the NMR data only in the C(14–17) site. That is, the 16(17)-epoxide, is replaced by a 2,3-dioxygenated tetrahydrofurane ring [an ethereal bridge between C-14 (δ_C_ 82.5 d) and C-17 (δ_C_ 82.1 d)] [61]. The epoxy-THF (C-12 to C-17) moiety of salarin J was found to be obtained by treating salarin A with MgBr_2_ as shown in Figure 14.

Finally, the earlier reported structure of the salarin B [56] was amended. The original structure implied one 12(13)-epoxide and a 16,17-diol. Indeed, the HRESIMS at *m/z* 703.2 was by 18 mu short, than anticipated [56,61] (Figure 11).

Worth mentioning are transannular NOE correlations between H-4 and H-13 observed for salarin-I, -C and -F and not for other salarins. It becomes clear that even small structural changes in the macrolide change its preferred conformation.

### 3.2. Tulearins A–C

**Figure 15:**
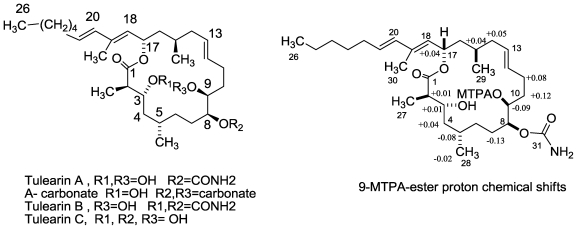
The structures of tulearins A–C, the cyclic 8,9-carbonate and MTPA-ester derivative.

The second group of isolated compounds composes the three tulearins, A–C [56,59] (Figure 15).

The HREIMS of tulearin A [56] exhibited a molecular ion [M + Na]^+^ at *m/z* 558.3757 proving, together with the carbon NMR spectrum, a formula of C_31_H_53_NO_6_Na, with six degrees of unsaturation. The structure of tulearin A was established by 1D and 2D NMR data [56]. Tulearin’s A core is a 2,4,15,19-tetramethylated hexaeicosanoic polyketide acid, possessing a 18 membered lactone (from C-1 to C-17), carrying on the macrolide chain, besides two hydroxyls (on C-3 and 9), also a carbamate (on C-8). The carbamate function is rare in nature, known for example in palmerolide A, recently isolated from a tunicate [72] or in the microorganism derived macrolide, geldanamycin [73].

Within the framework of a structure-activity relationship (SAR) study of the tulearins, we investigated the effects of a variety of reagents on the molecule in order to change the six functional moieties. Among others, we treated tulearin A with different bases in order to change the carbamate and/or the macrolide-lactone group, and also attempted to obtain tulearin C, *vide infra*, (Figure 15). Treatment of tulearin A with a mixture of aq ammonia/MeOH (1:1), afforded a less polar compound as colorless crystals in 83% yield. The structure of the latter compound was established to be the cyclic 8,9-carbonate derivative. Fortunately, the crystals of this carbonate were suitable for X-ray diffraction analysis, thus confirming tulearin‘s A structure and establishing the relative configuration of all seven chiral centers. The molecular structure of tulearin‘s A carbonate is depicted in Figure 15.

The absolute stereochemistry of tulearin A was determined by the modified Mosher‘s method [74]. The technique utilizes anisotropic shifts induced in the ^1^H NMR spectra of a-methoxy-a-(trifluoromethyl) phenylacetic (MTPA) esters of secondary alcohols to define the absolute configuration. Both (+)-(*R*)-(1*R*) and (−)-(*S*)-(1*S*) MTPA esters of tulearin A were prepared (Figure 15) and the Δδ values from their 500 MHz ^1^H NMR spectra were calculated Δδ [δ (S-MTPA ester) − δ(R-MTPA ester)]. Using this method, the absolute configuration of C-9 was determined to be *S*, hence, on the basis of the X-ray structure, the absolute configuration of the other chiral centers of tulearins A, B, and C (assuming the three to have a common biosynthesis) is 2*R*,3*R*,5*S*,8*S*,9*S*,15*R*, and 17*S* [59]. It is important to stress that, as required in the modified Mosher‘s method, all the assigned protons with positive and negative values are actually found on the right and left sides of the MTPA plane (MTPA-C-9 to C-4), respectively. Also, the absolute values of Δδ are inversely proportional to the distance from the MTPA moiety.

Two additional tulearins obtained from the *Fascaplysinopsis* sp. in very minor amounts were designated as tulearin B and C. Tulearin B was determined to be the 3,8-dicarbamate analog of tulearin A and tulearin C the 3,8,9-trihydroxy precursor of the tulearins [59].

### 3.3. Taumycin A and B

**Figure 16:**
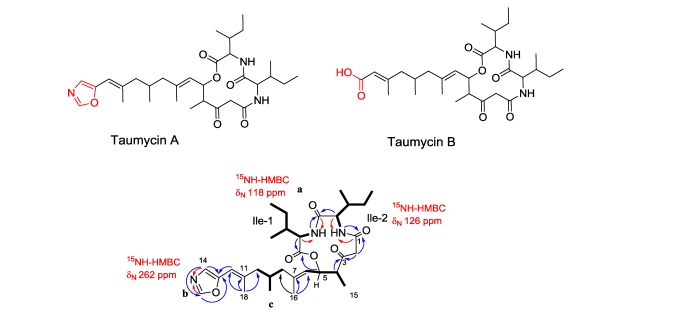
The structure of taumycin A and B and 2D correlations for taumycin A.

Together with the salarins and tulearins, we have isolated two closely related lipodepsipeptides named taumycins A and B (Figure 16) [58]. The HRCIMS of taumycin A revealed a pseudo molecular ion [M + H]^+^ at *m*/*z* 558.3559, corresponding to a molecular formula of C_31_H_47_N_3_O_6_ (Δ = 1.56 mmu) requiring 10 degrees of unsaturation. The structure of taumycin A was established from the ^1^H, ^13^C, COSY, HSQC, TOCSY, and HMBC spectra (Figure 16) [58], creating a 12-membered lipodepsipeptide. The attachment of the chain to the oxazole ring *i.e.*, to C-13 or C-14 was evidenced from *^1^J_CH_* values of CH(14) and CH(19), namely, 197 and 230 Hz, respectively. The latter values, measured from a Gated experiment, were compared to published values for 4- and 5-substituted oxazoles [75], hence, it could be concluded that the chain is attached to C-13 (the 5 position of the oxazole ring). The relative chirality of the four chiral centers of the depsipeptide was determined by Marfey‘s method [58]. The impossibility to distinguish between the position of the L and D Ile positions in the ring prevented assignment of the absolute configuration.

A 14-aminotetradecanoic moiety is known in nature, for example, in erythromycin; however, as the latter originates from seven molecules of propionate, there are, alternately, seven methyl groups in the molecule. In the taumycins, on the other hand, only three out of the four methyl groups are alternating.

Twelve-membered cyclic depsipeptides as in the taumycins are rare. Hapalosin, the first reported [76], as well as the recently reported acremolides A–D [77] and stereocalpin A [78], are all derived from microorganisms; the first from a cyanobacteria, the second from a marine-derived fungus, and the third from an Antarctic lichen. It is therefore very likely that the taumycins also derive from microorganism(s) living within the sponge. Taumycin A and B are toxic to brine shrimp larvae with IC_50_ values of 10 *μ*g/mL. Taumycin A, at 1 *μ*M, inhibited growth of the erythropoietin taumycin B as well as several derivatives of taumycin A which did not possess an antiproliferative effect on these cells.

### 3.4. Tausalarin C

**Figure 17:**
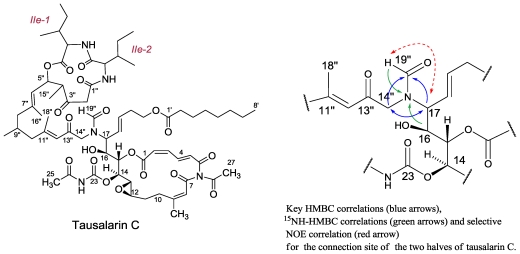
The structure of tausalarin C and connecting key 2D NMR correlations.

**Scheme 7:**
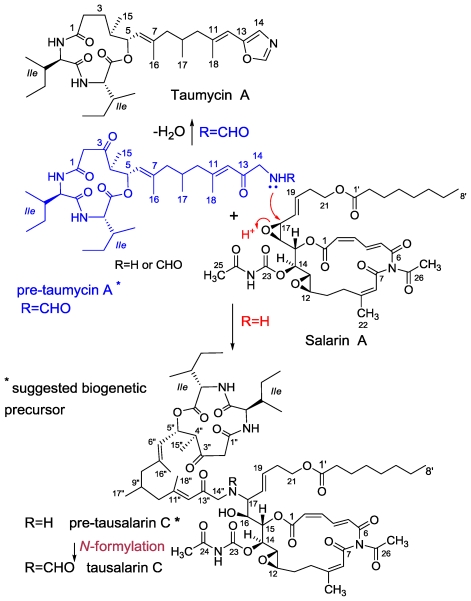
Suggested biogenesis on tausalarin C.

From three collections of the sponge, carried out in Salary Bay, in a depth of 25–35 m, we have isolated another metabolite designated tausalarin C (0.012% dry wet) (Figure 16) [60]. Spectral similarities of the NMR data of the latter compound to that of salarin A [56] and taumycin A [58], led us first to think that we were dealing with a 1:1 mixture of two compounds; however, unsuccessful HPLC separation efforts and mainly NMR correlations from one half of the molecule to the other, and at last, unmistakably, the HRESIMS spectrum, resulted in the slightly modified salarin A to taumycin A joint-structure. The mass spectroscopic analysis and ^13^C resonance values of tausalarin C, provided a pseudo molecular formula of C_66_H_95_N_5_O_19_Na in the positive HRESIMS mode, *m/z* 1284.6519, Δmmu 1.9 ppm, for [M + Na]^+^ and in the negative mode suitable [M − H]^−^ and [M + Cl]^−^, 1260.6586 and 1296.6310, Δmmu 1.5 ppm, peaks, respectively, implying 22 degrees of unsaturation. The ^1^H, ^13^C, COSY, HSQC, TOCSY, and HMBC spectra [60] established the conjugated structure of the compound as depicted in Figure 17. It became clear that the 16,17-epoxide of a salarin A molecule opened up by a nucleophilic amine attack. A bond from the latter nitrogen atom to C-17 of the opened epoxide moiety, of salarin A, connected the two parts of the molecule, one to the other, as established by the HMBC and NOE correlations completing the structure of tausalarin C (Figure 17) [60]. Interestingly, it was found that the 16,17-epoxide of salarin C can be opened, similarly to what is suggested for tausalarin C, selectively, with an amine and a suitable Lewis acid, to afford the 16-hydroxy-17-amino derivative (Scheme 7).

On the basis of the suggested biogenesis of tausalarin C (Scheme 7), the relative stereochemistry of the corresponding five stereogenic centers (C-12 to C-16) have to be the same as in salarin A and the chiral centers in the second half, the same as in taumycin A. The distance between the two halves of the molecule and the absence of meaningful NOE‘s between the two halves prevented the determination of the relative mutual stereochemistry between the two parts of the molecule.

The effect of tausalarin C on cell proliferation was determined in two different human leukemic cell lines, K562 [43] and UT7 [44] using the colorimetric methylthiazole tetrazolium bromide (MTT) assay [62] tausalarin C at 1 μM, inhibited 35%, 65% and 74% of K562 growth after 24 h, 48 h and 72 h, respectively—lower activity than measured for the separate two halves. Notably, tausalarin C did not significantly inhibit proliferation of the UT7 cells.

In addition to the four new groups of compounds we also isolated other known sponge metabolites, namely, a unique cyclitol glycolipid, crasseride [79] and several sterols including 5,8-peroxysterols, 9,11-secosterol carboxaldehyde [80].

## 4. Conclusions

The nitrogenous marine natural products presented above, describe only a few of the *N*-atom containing structures obtained from marine organisms. The wide scope of compounds, many of which possessing biological activities, exhibit the potential of the marine environment for novel structures and drug leads. Nature still seems to be without competition in the synthesis of novel outstanding compounds. The biogenesis of many of the new compounds is intriguing. While a biogenesis could be described for a few compounds like eilatin and violatinctamine, *vide supra*, the bio-synthesis of many others of the isolated compounds is debatable. In addition to the structure determination methodology, the review also reveals the problem of identifying the real source of the isolated compounds. Success in isolating microorganisms responsible for the synthesis of interesting compounds is of utmost importance for the supply of the latter compounds for biological tasks.
